# Using Static Multiple Light Scattering to Develop Microplastic-Free Seed Film-Coating Formulations

**DOI:** 10.3390/molecules29235750

**Published:** 2024-12-05

**Authors:** Rozenn Langlet, Romain Valentin, Marie Morard, Christine Delgado Raynaud

**Affiliations:** 1 Laboratoire de Chimie Agro-Industrielle (LCA), INRAE, Toulouse INP, Université de Toulouse, 31030 Toulouse, France; rozenn.langlet@ensiacet.fr (R.L.); romain.valentin@ensiacet.fr (R.V.); 2 Bois Valor—OLMIX, 13 Rue Jean Mermoz, 81160 Saint-Juéry, France; mmorard@boisvalor-olmix.com; 3 Centre d’Application et de Traitement des Agro-Ressources (CATAR), Toulouse INP, 31030 Toulouse, France

**Keywords:** microplastics, seed film-coatings, static multiple light scattering (SMLS)

## Abstract

Seed film-coatings used for seed treatment often contain microplastics which must be replaced. The objective of this study is to analyze the influence of substitutes (maltodextrin, waxy maize glucose syrup (WMGS), methylcellulose, tragacanth gum (TG), arabic gum (AG), polyvinyl alcohol (PVA), ethoxylated rapeseed oil (ERO)), and xanthan gum as a thickener on the stability of a seed film-coating via Static Multiple Light Scattering (SMLS) technology. The results demonstrate that the incorporation of each polymer results in an increase in the quantity of particles migrating from the supernatant phase, but a concomitant decrease in their sedimentation rate and in the thickness of the supernatant phase (e_c_). Furthermore, the redispersion capacity (*C_d_*) of the particles in the seed film-coating is also decreased after the introduction of each polymer, potentially due to their adsorption to the particles. The impact of the thickener is contingent upon the specific polymer employed. Its incorporation reduces the number of particles migrating from the supernatant phase and their sedimentation rate for all of the polymers studied except AG and ERO. However, it reduces e_c_ for all seed film-coatings. Depending on the substitutes, thickener incorporation either improves (WMGS, maltodextrin, AG) or deteriorates (TG, PVA, ERO) *C_d_*. The formulation containing tragacanth gum shows a redispersing capacity with *C_d_* ≤ 1. This study introduces a novel analytical criterion, the redispersion capacity *C_d_*, which can be employed to characterize dispersed systems.

## 1. Introduction

Seed treatment refers to the direct application of plant-protection products to the seed. This practice, which is commonly employed in contemporary agriculture, results in a reduction in the amount of plant-protection products used compared to foliar treatment [1].

Seed film-coatings are formulated products used to enhance the efficiency and safety of seed treatment. They fulfill several functions, such as (i) improving the adhesion of the seed treatment, (ii) reducing dust-off generated by treated seeds, which is rich in active substances, (iii) improving the seed flowability during sowing, resulting in better sowing precision, (iv) reducing the seed treatment leaching and controlling the release of active ingredients, and (v) identifying treated seeds and improving their visual appearance.

To perform these functions, seed film-coatings typically contain dispersed polymer particles, which can be a source of microplastic generation in the environment. These polymers therefore contribute to microplastic pollution [2] during the production process (unintentional release), potentially during the use phase (the generation of microplastic dust [3]), and at the end-of-life phase (soil fragmentation).

According to the European Chemicals Agency (ECHA), 0.22% of the manufactured microplastics released into the environment in the European Union annually come from seed film-coatings, i.e., 500 tons [4].

In this context, it is therefore necessary to find substitutes for these polymers, which can often be acrylics [1], which are not microplastics and do not generate microplastics during their use and end-of-life. Accinelli et al. [3,5] were the first to mention the problem of microplastics in seed film-coatings and explicitly propose a substitute; then, in 2024, Langlet et al. [1] published a review on the topic.

Incorporating these substitutes into formulations requires their modification or even the development of new formulations to maintain their performance. To this end, it is therefore crucial to be able to understand and assess the stability of these formulations, which are often multi-phase systems. To date, no study on this subject has been identified in the scientific literature.

Analytical techniques are available for the study of dispersion stability, such as zetametry [6,7] and dynamic light scattering (DLS) analysis [6,7], which require samples with low-concentration, or optical or electron microscopy [7,8], which permit only a local observation of the sample under study. Static Multiple Light Scattering (SMLS) technology offers the advantage of not requiring the dilution of the samples, while simultaneously enabling the observation of any destabilization throughout the sample [9]. This technology has been employed in a multitude of works to examine the stability of dispersions [9,10,11] and emulsions [12,13,14]. The majority of studies employs the Turbiscan device, and the Turbiscan Scan Index (TSI) [10,11,13,15], a criterion integrated into the TurbiSoft Classic 2 software (2.3.1.125, FAnalyser 1.3.5) dedicated to investigate the stability of multiphase systems. This criterion enables the quantitative assessment of stability across the entire sample set, facilitating comparisons between multiple samples. However, its use alone does not account for the type of destabilization observed, nor for the ease of sample redispersion. Other studies focus on destabilization kinetics [9] or the evolution of scans over time [14]. In this study, we introduce a new criterion useful to the elaboration of new microplastic-free seed film-coating formulations, namely redispersion capacity, based on the mean value of backscattered light intensity, BS, in the entire sample at t = 0 and after accelerated aging at 54 °C, followed by redispersion.

In this study, we endeavor to elucidate the impact of the incorporation of specific polymers, which may be regarded as substitutes for the microplastics actually used in seed film-coatings, as they meet the requirements of the REACH regulation [1], and of a thickener (xanthan gum) on the stability of a model seed film-coating formula, employing SMLS technology. A hitherto unreported study criterion, redispersion capacity, was calculated using SMLS data.

## 2. Results

Seed film-coatings typically comprise mineral particles or pigments to perform the functions of identifying treated seeds and improving their visual appearance. These particles, insoluble in water, eventually sediment, but may be stabilized by surfactants and polymers. Any change in a polymer in the sample thus entails a modification of the seed film-coating’s stability. It is then necessary to study the stability of seed film-coatings for each polymer substitution with and without the addition of a thickener (xanthan gum; here referred to as pregel), used to stabilize the formulation. The stability of the formulas investigated was studied using SMLS technology, and their aging was accelerated by increasing their storage temperature to 54 °C during 2 weeks.

### 2.1. Identifying the Type of Destabilization

The device measures BS over the entire tube (one measurement every 20 µm) at a given time. By subtracting the scans obtained over time from the reference scan (chosen at t = 0), it is possible to identify the type of destabilization that is occurring.

The values displayed subsequently correspond to Δ*BS*, the variation in backscattered light flux. Please refer to the Supplementary Materials for further details (Figure S1). Photographs of two samples (M14.5 and M14.5 + P) during the accelerated aging are also shown in the Supplementary Materials (Figure S2).

A notable decline in backscattering was observed at the upper portion of the tube for all samples, which is indicative of clarification. The reduction in particle concentration at the upper portion of the tube would typically be accompanied by an increase in the remaining regions, resulting in a theoretical variation in BS in the rest of the tube. However, this variation is not observed for any sample.

This may be attributed to an excessively high local particle volume fraction. The particle volume fraction can be calculated to range between 10 and 14% based on the density of the seed film-coatings studied (between 1 and 1.4) and that of the suspended particles. Therefore, as sedimentation occurs at the bottom of the tube, the local volume fraction may approach the critical volume fraction, at which point backscattering no longer varies according to the particle volume fraction. Consequently, in the remainder of this study, in the event of clarification, our focus will be sorely on the observed decrease in particle number observed at the tube’s apex.

### 2.2. Impact of Susbtitute Addition on Seed Film-Coating Stability

**Figure 1:**
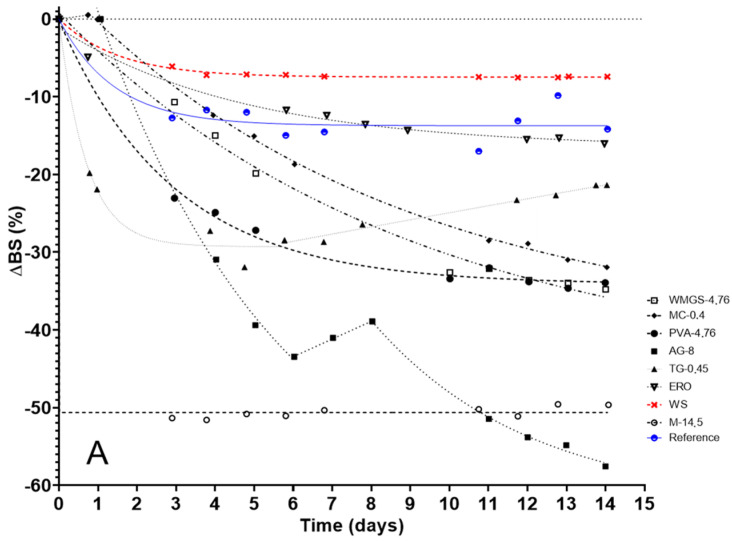

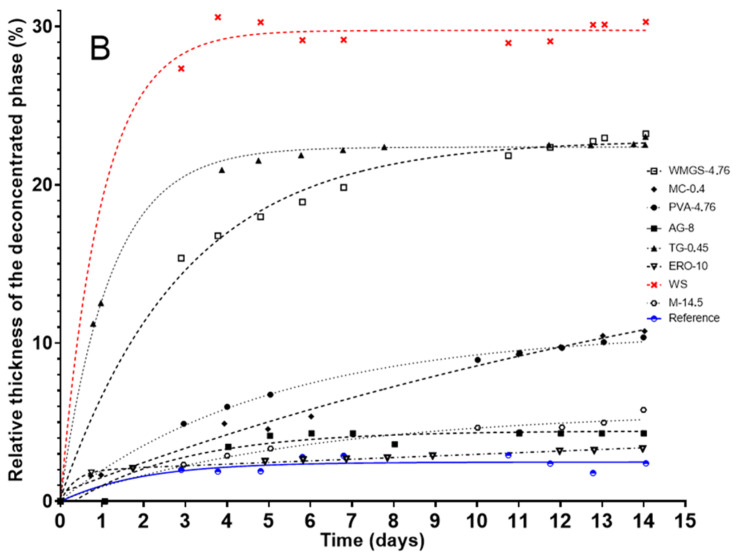
The kinetics of the mean backscatter loss Δ*BS_average_* at the top of the tube (**A**) and the relative average thickness of the supernatant phase e_c_ (**B**), as a function of time.

In order to investigate the impact of the introduced substitute, a time-series analysis was conducted on the average backscattering loss at the upper end of the tube Δ*BS_average_*, as well as the relative average thickness of the supernatant phase e_c_ (Figure 1).

In all samples, the backscattered light intensity Δ*BS* decreases exponentially over time, with the exception of TG-0.45 and AG-8. For TG-0.45, Δ*BS* exhibits an exponential decrease to −31.93% during the first five days of accelerated aging, followed by a linear rise to reach −21.36% after 14 days of accelerated aging. For AG-8, Δ*BS* decreases exponentially from one day of accelerated aging to −43.44% after six days of aging, then rises again to −38.89% over the following two days. On day 8 of aging, Δ*BS* again decreases exponentially until day 14 of aging (−57.55%).

With regard to the relative mean thickness of the supernatant phase, it increases at a slower and less pronounced rate when the seed film-coating contains a substitute. The exponential increase in e_c_ was observed for all samples, with the exception of substitute ERO-10, where e_c_ exhibited an initial exponential increase up to two days of aging, followed by a linear increase.

The impact of polymer addition on formulation stability can be discerned by comparing the destabilization parameters of the formulation with those of the model formulation devoid of a substitute or pregel (Table 1).

The destabilization parameters used for the study are explained further in 4.4. The destabilization of the seed film-coating is an acceptable criterion, provided that it is reversible following agitation. To monitor this criterion, the samples are vortexed at the end of accelerated aging process at a speed (544 rpm) equivalent to that found in industry, and the redispersion capacity *C_d_* is calculated. Mathematically, a *C_d_* value ≤ 1 corresponds to a dispersion state equivalent to the one before accelerated aging. It could be up to the industrial formulator to increase this threshold of 1 according to the respective industrial specifications.

The incorporation of substitutes into the formula resulted in a reduction in final Δ*BS_average_* for all substitutes under investigation. Consequently, following the accelerated aging process, the supernatant phase exhibited a diminished concentration of particles. Conversely, the time required for clarification to become apparent is longer. The final relative thickness of the supernatant phase e_f_ is lower, following the addition of the aforementioned substitutes. Finally, the average sedimentation rate V_average_ is also significantly reduced by the addition of the substitutes. However, the incorporation of substitutes leads to an increase in *C_d_* ≥ 1, which translates into a redispersion that does not return to its initial state.

The ERO-10 substitute exhibits the lowest Δ*BS_average_* reduction and the highest e_f_ reduction. Additionally, it is also the seed film-coating with the lowest V_average_. However, its redispersion capacity is above 1 (1.72), indicating that it is difficult to redisperse the material to its original state at the end of accelerated aging. The tragacanth-containing seed film-coating exhibits the best redispersion capacity (1.10), although it remains above 1, suggesting that the redispersed state is not identical to the initial state.

Ultimately, following a comparative analysis of the destabilization parameters of the seed film-coatings, incorporating the substitutes with those of the reference seed film-coating, it can be concluded that no seed film-coating exhibits *C_d_* ≤ 1 except the seed film-coating containing tragacanth gum, which displays redispersion capacity. The seed film-coating containing ethoxylated rapeseed oil also exhibits a comparatively lower average sedimentation rate.

### 2.3. Influence of Pregel Addition on Film Stability

**Figure 2:**
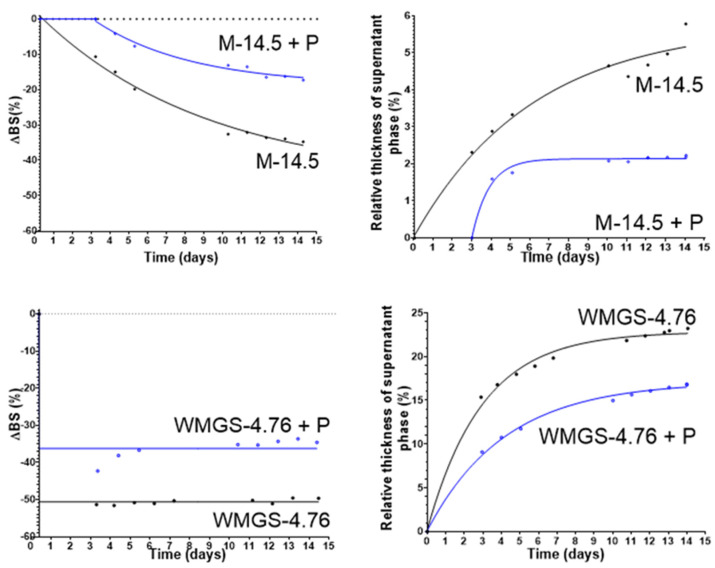

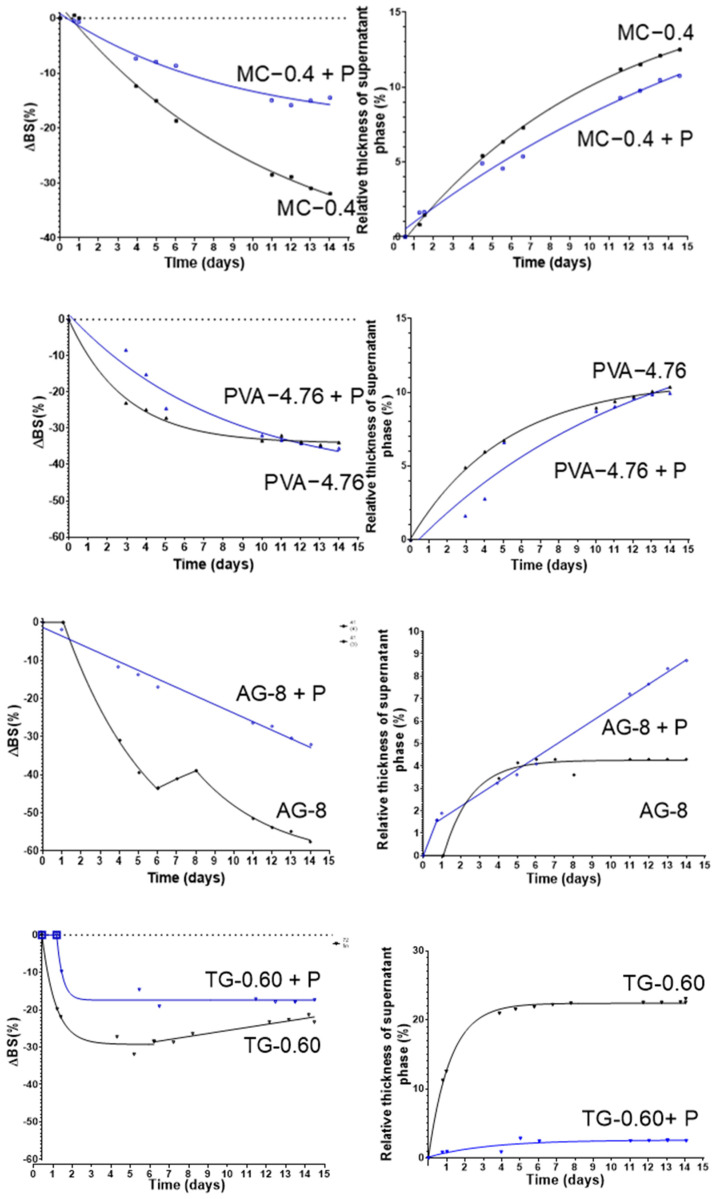

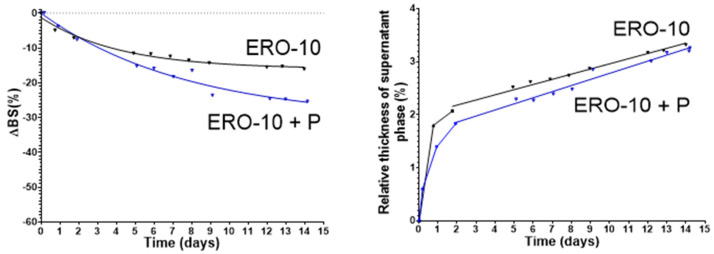
The destabilization kinetics of the substitute-containing seed film-coatings, with and without pregel. **Left**: the kinetics of the backscattering loss Δ*BS_average_*; **right**: the kinetics of the relative mean thickness of the supernatant phase e_c_. The black line corresponds to the seed film-coating formulations without pregel, and the blue one to the ones with pregel.

To assess the impact of thickener incorporation on the samples, we conducted a time-series analysis of the average backscatter loss Δ*BS_average_* at the apex of the tube, and the relative average thickness of the supernatant phase e_c_ (Figure 2).

The curves corresponding to pregel-containing seed film-coatings are shown in blue for all investigated substitutes. Δ*BS_average_* is higher for seed film-coating formulations containing pregel, except for PVA-4.76 and ERO-10. A higher Δ*BS* means that the addition of pregel reduces the number of particles migrating from the supernatant phase.

After 12 days of aging, the Δ*BS* of PVA-4.76 + P is lower than that of PVA-4.76. For ERO-10, the Δ*BS* of ERO-10 + P is lower after 5 days of aging. For most of the samples, with and without pregel, an exponential decrease in Δ*BS* is observed, as the particles’ volume concentration (*Φ*) and average diameter (*d*) at the tube apex decrease rapidly at first, then more slowly.

The addition of pregel does alter the exponential decay of Δ*BS* in samples containing Arabic gum (AG) and tragacanth gum (TG). For Arabic gum, Δ*BS* decreases linearly after the addition of pregel, and for tragacanth gum, an initial exponential decrease is observed, followed by a linear increase in Δ*BS*. These changes suggest that the evolution speed of d and *Φ* is affected by the incorporation of the pregel. It is possible that for these polymers, xanthan gum, alone or in combination with the substitute, adsorbs onto the particles, increasing their diameter and making the particle size distribution more uniform. This could explain the shift from an exponential to a linear trend.

The addition of pregel to the seed film-coating reduces the relative thickness of the supernatant phase e_c_ for all seed film-coatings studied, except the one containing Arabic gum, where e_c_ is greater after 6 days of accelerated aging. The addition of pregel does not alter the trend of e_c_ evolution except for Arabic gum, where e_c_ evolves linearly rather than exponentially after pregel addition.

The parameters measured and calculated as described in the Section 4 are shown in Table 2.

For all seed film-coatings studied, the addition of pregel reduces V_average,_ except for the seed film-coating containing Arabic gum and ethoxylated rapeseed oil, where it slightly increases. Depending on the substitutes, the addition of pregel to the seed film-coating either improves (waxy corn glucose syrup, maltodextrin, Arabic gum) or deteriorates (tragacanth gum, polyvinyl alcohol, ethoxylated rapeseed oil) its redispersion capacity.

The seed film-coating containing ethoxylated rapeseed oil without pregel exhibits the highest final Δ*BS_average_*, and the lowest e_f_ and V_average_. On the other hand, the seed film-coating containing tragacanth gum with pregel has the best redispersion capacity, followed by the seed film-coating containing maltodextrin with pregel.

When compared to the reference seed film-coating, only TG-0.6 has a satisfactory redispersion capacity. Although the seed film-coatings M-14.5 + P, TG-0.6 + P and MC-0.4 + P have either satisfactory e_f_ or final Δ*BS_average_*, their redispersion capacity is insufficient, meaning that their destabilization is not reversible.

## 3. Discussion

### 3.1. Influence of Substitute Addition on Seed Film-Coating Stability

#### 3.1.1. Maltodextrin and Waxy Maize Glucose Syrup

The addition of maltodextrin and waxy corn glucose syrup reduces the final Δ*BS_average_*, e_f_, and V_average_. The redispersion capacity of seed film-coatings is also deteriorated. More particles migrate, but at a slower rate. It can be assumed that the more numerous particles form aggregates as they migrate, which are more difficult to redisperse but are stabilized by the increased viscosity of the continuous phase provided by the polymer.

It has been reported in the literature that dextrin can adsorb to the surface of mineral particles via hydrogen bonding and chemical interactions with the metal hydroxides on their surface [16]. It is therefore possible that the adsorbed molecules cause the particles to agglomerate more easily than the talc particles contained in the adhesive-free seed film-coating, which can repel each other via their zeta potential [17]. Consequently, the redispersion of seed film-coatings formulated with starch derivatives is more difficult.

#### 3.1.2. Methylcellulose

The addition of methylcellulose decreases the final Δ*BS_average_*, e_f_, and V_average_. Additionally, the redispersion capacity of the seed film-coating is deteriorated. It is likely that the sedimentation rate is diminished due to the increased viscosity of the continuous phase resulting from the introduction of methylcellulose. With respect to redispersion capacity, it is conceivable that methylcellulose may facilitate irreversible particle agglomeration.

#### 3.1.3. Polyvinyl Alcohol

The addition of polyvinyl alcohol reduces the final Δ*BS_average_*, e_f_, and V_average_. The redispersion capacity of the seed film-coating is deteriorated.

One study [18] reports the adsorption of polyvinyl alcohol chains on the surface of particles composed of a mixture of Al O_23_ -SiO_2_ -TiO_2_. At pH 6, the overall charge on the particle surface is nearly zero, with a comparable number of local negative and positive charges. Hydrogen bonds can be formed between polymer chains and hydroxyl groups on the particle surface. The adsorption of these polymers stabilizes the suspension, resulting in a reduction in the intensity of clarification observed at the top of the tube. The adsorbed particles are sterically stabilized, and the authors hypothesize the formation of single polymer bridges between the adsorbed chains on two different particles. This hypothesis could explain the significant reduction in redispersion capacity observed.

#### 3.1.4. Arabic Gum

The addition of arabic gum reduces the Δ*BS_average_*, e_f_, and V_average_. The redispersion capacity of the seed film-coating is deteriorated.

The sedimentation rate may be reduced by the viscosity increase in the seed film-coating, due to the arabic gum chains.

Arabic gum has the capacity to adsorb onto the surface of metal oxides such as TiO_2_ [19]. At pH = 7, the substance would have no effect on the zeta potential of the particles, but would enable steric repulsion between them. The authors propose that a bridging phenomenon may also occur between particles, resulting in a slight flocculation of the particles and the formation of peaks observed in the middle of the tube (Supplementary Materials). It is plausible that this flocculation is not entirely reversible, which could explain the reduced redispersion capacity.

#### 3.1.5. Tragacanth Gum

The addition of tragacanth reduces the final Δ*BS_average_*, e_f_, and V_average_. The redispersion capacity of the seed film-coating is improved.

One study demonstrated that tragacanth has the capacity to adsorb onto the surface of mineral particles [20]. As this occurs, tragacanth causes a slight increase in the zeta potential of the particles, thereby reducing their electrostatic repulsion. However, the study did not address the potential for steric repulsion to occur.

#### 3.1.6. Ethoxylated Rapeseed Oil

The addition of ethoxylated rapeseed oil reduces the final Δ*BS_average_*, e_f_, and V_average_. The redispersion capacity of the seed film-coating is deteriorated.

As a surfactant, it is conceivable that ethoxylated rapeseed oil may adsorb to the surface of particles such as talc, thereby stabilizing the dispersion through steric repulsion.

### 3.2. Influence of Pregel Addition on Film Stability

The addition of pregel to all seed film-coatings under study resulted in an increase in the final Δ*BS_average_*, and a decrease in V_average_, meaning that the number of particles migrating from the supernatant phase and their migration speed were reduced, except for the seed film-coating containing arabic gum and ethoxylated rapeseed oil.

Nevertheless, the addition of pregel diminished e_f_, i.e., the thickness of the supernatant phase, for all seed film-coatings.

The addition of pregel to the seed film-coating affects the redispersion capacity of the seed film-coating, depending on the substitute used. In some cases, the capacity is improved (waxy corn glucose syrup, maltodextrin, arabic gum), while in others, it is deteriorated (tragacanth, polyvinyl alcohol, ethoxylated rapeseed oil).

A number of hypotheses can be proposed regarding the influence of the pregel. The pregel consists of xanthan gum, a polymer that has been the subject of extensive research in the literature [21,22,23,24]. In light of the available literature, the following hypotheses can be put forth:

- The addition of xanthan gum in solution has been observed to increase the viscosity of the continuous phase at rest. This observation is consistent with the predictions of Stokes’ law, which suggests that the addition of the gum should slow gravity-induced sedimentation.
- Xanthan gum may also slow down sedimentation by adsorption to the surface of mineral particles such as talc or TiO_2_. This adsorption may lead to a possible stabilization of these particles, present in the model seed film-coating, via electrostatic [21] and steric [22] repulsion phenomena.

It is possible that the addition of pregel in seed film-coatings containing arabic gum or ethoxylated rapeseed oil results in less adsorption of arabic gum or ethoxylated rapeseed oil chains to the surface of TiO_2_ particles. The stabilizing effect of xanthan gum is likely to be less significant than that of arabic gum or ethoxylated rapeseed oil.

The difference in impact of the pregel addition on the redispersion capacity of substitute-containing seed film-coatings could be attributed to the capacity of the polymer chains adsorbed on the particles to either facilitate, or, conversely, complicate, particle agglomeration in comparison to the xanthan gum chains contained in the seed film-coating with pregel, which also adsorb to the particles and can repel each other via their zeta potential [17].

## 4. Materials and Methods

The following compounds have been studied in the seed film-coating: maltodextrin (DE = 17, Glucidex 17, Roquette, Lestrem, France), tragacanth gum (Tragacanth powder, Alfa Aesar, Ward Hill, MA, USA), methylcellulose (Methycellulose, viscosity 1600 cPs, Alfa Aesar, Ward Hill, MA, USA), waxy maize glucose syrup (N-tack, Ingredion, Westchester, IL, USA), polyvinyl alcohol (Poval 3:85, Kuraray, Chiyoda, Japan), Arabic gum (Instant Gum BC, Nexira, Rouen, France), and ethoxylated rapeseed oil (Sepigreen C300, Seppic, Paris, France). The seed film-coating was prepared using a Digital Overhead Stirrer (DLS), and a deflocculator-type paddle, by stirring at 500 rpm for 30 min.

### 4.1. Composition of the Formulations

The studied formulations are aqueous colored dispersions, whose proportions of substitute polymer and pregel are detailed in Table 3. The proportion of pregel in formulas was determined based on viscosity requirements (300–1000 cPs).

### 4.2. Accelerated Aging

In order to study the stability of the seed film-coating over time, its long-term aging was simulated by storing it for 2 weeks at 54 ± 2 °C, according to the conditions of the CIPAC MT method 46 [25], an accelerated aging procedure used for plant-protection product formulations. According to this method, 2 weeks of storage at 54 °C is equivalent to 2 years of storage at room temperature.

### 4.3. Stability Measurement with SMLS

The influence of the addition of each substitute and the thickener on the seed film-coating’s stability is investigated, by comparing the stability of each formula (Table 3) to the stability of a formula containing no substitute nor thickener (WS in Table 3). The stability of the formulas is also compared to the reference (Ref in Table 3) containing microplastics, whose stability is known and satisfactory.

Formula stability is monitored using the Turbiscan Classic 2 (Microtrac, Montgomeryville, PA, USA), which uses SMLS to detect stability changes in dispersed systems such as emulsions, dispersions, and foams. Samples are homogenized at room temperature for 2 min using a magnetic stirrer. A total of 7 mL of sample is transferred to each Turbiscan measuring tube (inner diameter = 5 mm) and then subjected to accelerated aging at 54 °C for two weeks. A first scan is performed at t = 0 after homogenization; then, the samples are removed from the oven for scanning every 24 h.

#### 4.3.1. SMLS Principle

In liquid dispersions, SMLS technology enables the detection and monitoring of particle migration and agglomeration through the measurement of backscattering light (*BS*), which is influenced by the local particle volume fraction (*Φ*) and diameter (*d*). When *Φ* exceeds the critical particle volume fraction (*Φ_c_*), the system enters a concentrated regime where *BS* intensity can be approximated by (1)

(1) $$BS\approx \surd (\frac{3\Phi \left(1-g\right)Qs}{2d})$$

With *BS* the backscattered light intensity in %,
*d* the particle diameter in µm,
*Φ* the volume fraction of the dispersed phase,
and *g* (asymmetry factor) and *Qs* (scattering efficiency) derived from Mie’s theory.

Around the saturation particle volume fraction *Φs* (8–30%), the intensity of backscattered light varies very little with the particle volume fraction, then decreases as the particle volume fraction increases.

#### 4.3.2. Study Criteria

Several parameters can be recorded or calculated to study the stability of the seed film-coating under study. In fact, the seed film-coating studied is a polyphasic system in which particles are dispersed into the media. The parameters measured or calculated for the study of seed film-coating stability are as follows:

- The onset of seed film-coating destabilization by clarification;
- The average difference in backscattered light flux in the supernatant phase between the final and initial states (Final Δ*BS_average_*);
- The time taken to reach Final Δ*BS_average_*;
- The relative thickness of the supernatant phase in the final state (e_f_);
- The time taken to reach e_f_;
- The average sedimentation velocity of the particles and/or droplets (V_average_);
- The redispersion capacity *C_d_*.

The phase-shift kinetics are obtained from TurbiSoft Classic 2 (2.3.1.125, FAnalyser 1.3.5), using the “Advanced calculations/Average value” and “Advanced calculations/Peak width” functions at the top of the tube. The threshold for the peak width function is set to Δ*BS* = −2%. The sedimentation rate is obtained from the derivative of the mathematical expression describing the kinetics of the evolution of the phase shift front (kinetics derived from the “Peak width” function).

At the end of the aging period, the samples are vortexed at 544 rpm for 1 min and then re-analyzed with the Turbiscan to assess the reversibility of the destabilization by agitation. The redispersion capacity is calculated according to the following formula:

$$C_{d}=\frac{{\left(\frac{1}{N_{h}}\sum_{h=h_{max}}^{h=0}\frac{\left|BS(h)-{BS}_{average}\right|}{{BS}_{average}}\right)}_{t=0}}{{\left(\frac{1}{N_{h}}\sum_{h=h_{max}}^{h=0}\frac{\left|BS(h)-{BS}_{average}\right|}{{BS}_{average}}\right)}_{\;after\;redispersion}}$$

With

*N_h_* = the number of measurements made by the Turbiscan along the tube, corresponding to the height of the sample divided by 20 µm;
*h_max_* = total height of seed film-coating in tube at *t* = 0;
*BS* (*h*) = the percentage of light backscattered at a height *h*;
*BS_average_* = the average percentage of backscattered light between *h* = 0 and *h* = *h_max_*.

## 5. Conclusions

The purpose of this study was to investigate the effect of adding specific polymers and xanthan gum to a seed film-coating formulation, using SMLS technology. This study showed that the addition of a polymer increases the number of particles migrating from the supernatant phase, but decreases their sedimentation rate and the thickness of the supernatant phase. The redispersion capacity of the particles contained in the seed film-coating is also lower after the addition of polymers, possibly due to their adsorption to the particles. These observations are independent of the polymer studied.

The effect of xanthan gum differs according to the polymer added; its addition reduces the number of particles migrating from the supernatant phase, and their migration speed for all the polymers studied except for arabic gum and ethoxylated rapeseed oil. However, the addition of pregel reduces the thickness of the supernatant phase for all seed film-coatings. Depending on the substitutes, the addition of pregel to the seed film-coating either improves (waxy maize glucose syrup, maltodextrin, arabic gum) or deteriorates (tragacanth gum, polyvinyl alcohol, ethoxylated rapeseed oil) its redispersion capacity. The formulation containing tragacanth gum showed redispersing capacity with *C_d_* ≤ 1_,_ which seems promising for the substitution of microplastics in seed film-coating, although its performances need to be assessed.

The redispersion capacity *C_d_* used in this study is a criterion calculated from SMLS Turbiscan data that has never been mentioned before and may be useful for the study of dispersed systems.

## Figures and Tables

**Table 1:** The destabilization parameters of the substitute-containing seed film-coatings, the reference film seed-coating, and the model film seed-coating without a substitute or pregel. Bold *C_d_* ≤ 1.

Formula Composition	Start of Clarification	Final Δ*BS_average_*	Time to Reach Final Δ*BS_average_*	e_f_ (%)	Time to Reach e_f_	V_average_ (%/day)	*C_d_*
Reference	3 days	−14.17%	3 days	2.40%	3 days	0.21	**0.96 ± 0.05**
WS	4 days	−7.40%	4 days	30.29%	4 days	3.17	**0.51 ± 0.03**
WMGS-4.76	3 days	−49.37%	3 days	23.22%	14 days	1.77	2.58 ± 0.07
M-14.5	3 days	−34.77%	14 days	5.78%	14 days	0.37	2.91 ± 0.01
MC-0.4	1 day	−31.94%	14 days	12.50%	14 days	0.90	4.36 ± 0.20
TG-0.6	1 day	−23.32%	4 days	23.04%	4 days	2.19	**0.65 ± 0.03**
PVA-4.76	3 days	−57.55%	14 days	4.29%	5 days	0.21	1.69 ± 0.08
AG-8	1 day	−33.92%	10 days	10.36%	14 days	0.74	2.29 ± 0.14
ERO-10	1 day	−16.06%	14 days	3.32%	14 days	0.10	1.72 ± 0.10

WS = without substitute, WMGS = waxy maize glucose syrup, M = maltodextrin, MC = methylcellulose, TG = tragacanth gum, PVA = polyvinyl alcohol, AG = arabic gum, ERO = ethoxylated rapeseed oil. Numbers correspond to the substitute’s mass proportion in the formulation. e_f_ = relative thickness of the supernatant phase in the final state; V_average_ = the average sedimentation velocity of the particles and/or droplets; *C_d_* = the redispersion capacity, which must be ≤ 1.

**Table 2:** Destabilization parameters of seed film-coatings containing substitutes with and without pregel. Bold *C_d_* ≤ 1.

Formula Composition	Start of Clarification	Final Δ*BS_average_*	Time to Reach Final Δ*BS_average_*	e_f_ (%)	Time to Reach e_f_	V_average_ (%/day)	*C_d_*
Reference	3 days	−14.17%	3 days	2,40%	3 days	0.21	**0.96 ± 0.05**
WMGS-4.76	3 days	−49.37%	3 days	23.22%	14 days	1.77	2.58 ± 0.07
WMGS-4.76 + P	3 days	−34.58%	3 days	16.84%	14 days	1.00	1.11 ± 0.02
M-14.5	3 days	−34.77%	14 days	5.78%	14 days	0.37	2.91 ± 0.01
M-14.5 + P	4 days	−17.31%	14 days	2.23%	14 days	0.22	**0.68 ± 0.04**
MC-0.4	1 day	−31.94%	14 days	12.50%	14 days	0.90	4.36 ± 0.20
MC-0.4 + P	1 day	−14.47%	14 days	10.75%	14 days	0.74	ND
TG-0.6	1 day	−23.32%	4 days	23.04%	4 days	2.19	**0.65 ± 0.03**
TG-0.6 + P	1 day	−17.37%	3 days	2.47%	4 days	0.19	1.44 ± 0.09
PVA-4.76	3 days	−57.55%	14 days	4.29%	5 days	0.21	1.69 ± 0.08
PVA-4.76 + P	1 day	−32.05%	14 days	8.70%	14 days	0.54	2.02 ± 0.15
AG-8	1 day	−33.92%	10 days	10.36%	14 days	0.74	2.29 ± 0.14
AG-8 + P	1 day	−35.53%	14 days	9.93%	14 days	0.78	2.18 ± 0.13
ERO-10	1 day	−16.06%	14 days	3.32%	14 days	0.10	1.72 ± 0.10
ERO-10 + P	1 day	−24.71%	14 days	3.20%	14 days	0.12	1.81 ± 0.06

WS = without substitute, WMGS = waxy maize glucose syrup, M = maltodextrin, MC = methylcellulose, TG = tragacanth gum, PVA = polyvinyl alcohol, AG = arabic gum, ERO = ethoxylated rapeseed oil. Numbers correspond to the substitute’s mass proportion in the formulation. e_f_ = relative thickness of the supernatant phase in the final state; V_average_ = the average sedimentation velocity of the particles and/or droplets; *C_d_* = the redispersion capacity, which must be ≤ 1. ND corresponds to not determined.

**Table 3:** The proportions of polymer and pregel in the studied seed film-coatings. The formulas correspond to the polymer and non-described ingredients.

Name	Polymer	Mass of Product (g) per 100 g of Formula	Mass of Pregel (g) per 100 g of Formula
Ref	Microplastic	16.5	5.00
WS	/	/	0
M-14.5	Maltodextrin	14.50	0
M-14.5 + P		14.50	2.50
WMGS-4.76	Waxy maize glucose syrup	4.76	0
WMGS-4.76 + P		4.76	2.50
TG-0.6	Tragacanth gum	0.60	0
TG-0.6 + P		0.60	2.50
MC-0.40	Methylcellulose	0.40	0
MC-0.40 + P		0.40	2.50
PVA-4.76	Polyvinyl alcohol	4.76	0
PVA-4.76 + P		4.76	2.50
AG-8	Arabic gum	8.00	0
AG-8 + P		8.00	2.50
ERO-10	Ethoxylated rapeseed oil	10.00	0
ERO-10 + P		10.00	2.50

## Data Availability

Data available on request due to restrictions (privacy reasons).

## Supplementary Materials

The following supporting information can be downloaded at: https://www.mdpi.com/article/10.3390/molecules29235750/s1, Figure S1: Delta backscattering profiles (Δ*BS*) of seed film-coating formulas under study. Figure S2: Photographs of M14.5 (up) and M14.5 + P (down) during the accelerated aging.
