# Supplementary material for: Using Static Multiple Light Scattering to Develop Microplastic-Free Seed Film-Coating Formulations
Source: Molecules. 2024 Dec 5;29(23):5750. doi: 10.3390/molecules29235750 (PMC11643206; doi:10.3390/molecules29235750)
Supplement: Supplementary file 1 [file molecules-29-05750-s001.zip › molecules-3262872-supplementary.pdf]

Delta Backscattering – Ref

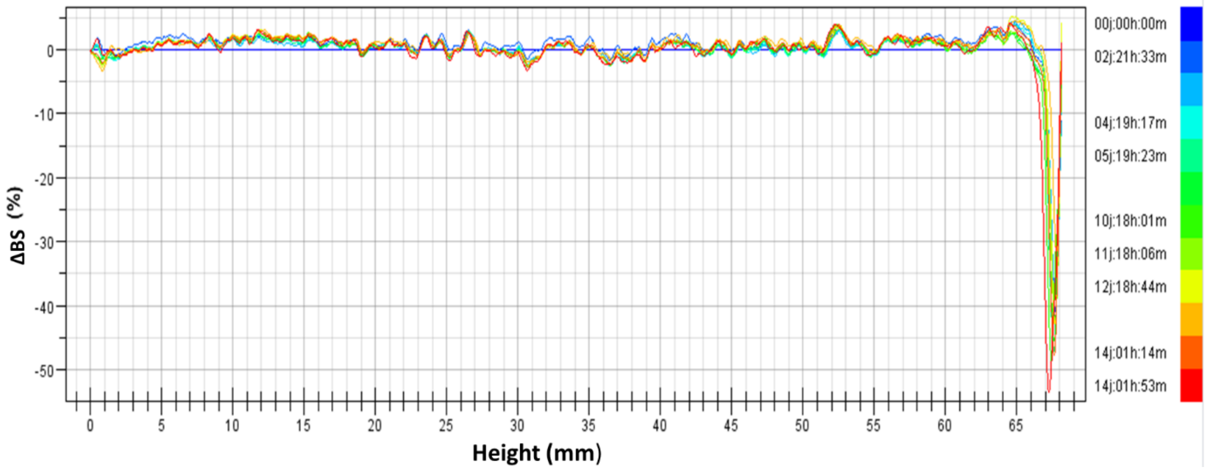

Delta Backscattering – M-14,5

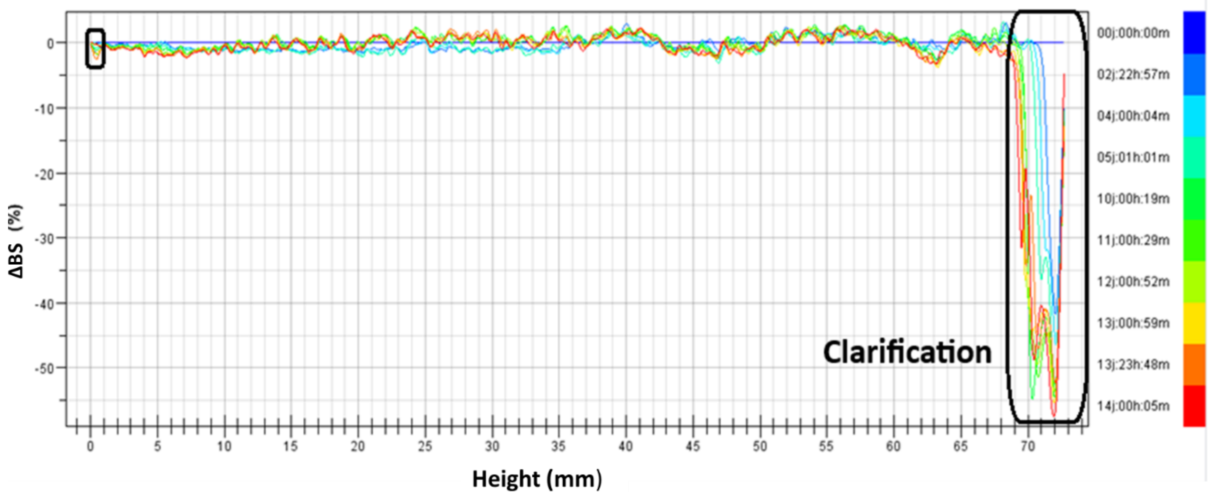

Delta Backscattering – WS

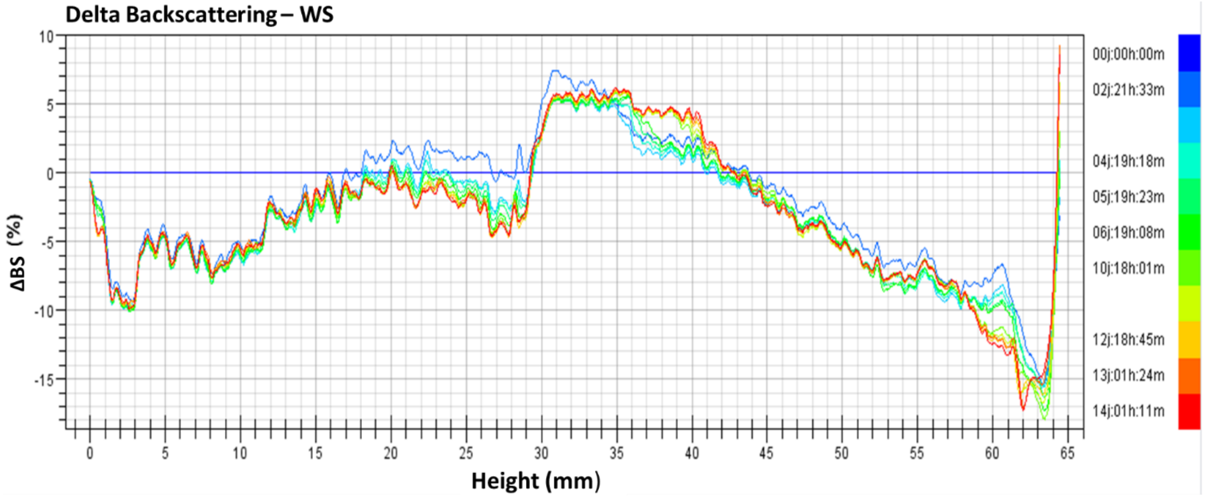

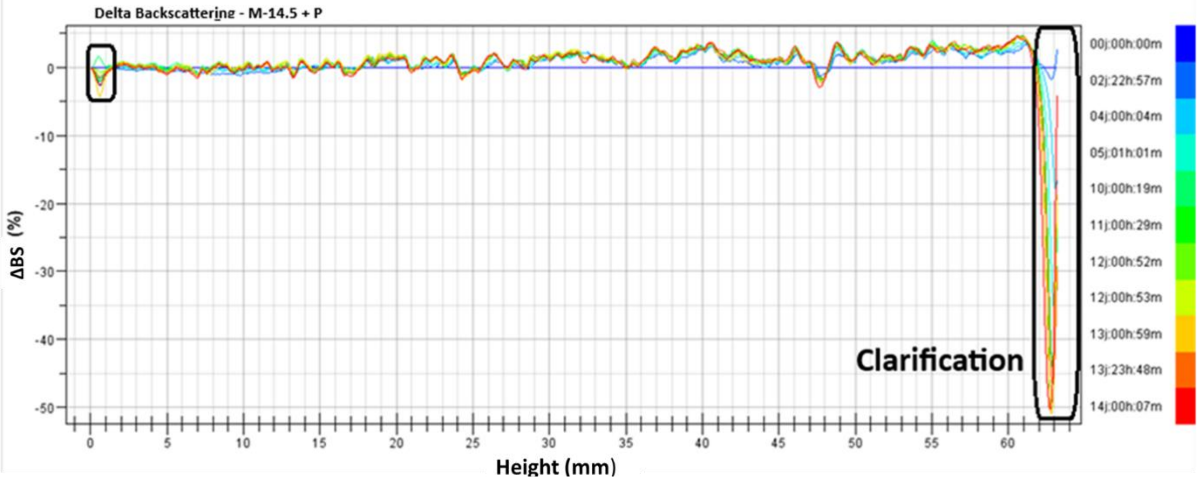
Delta Backscattering - M-14.5 + P
ΔBS (%)
Height (mm)
Clarification
00j:00h:00m
02j:22h:57m
04j:00h:04m
05j:01h:01m
10j:00h:19m
11j:00h:29m
12j:00h:52m
12j:00h:53m
13j:00h:59m
13j:23h:48m
14j:00h:07m

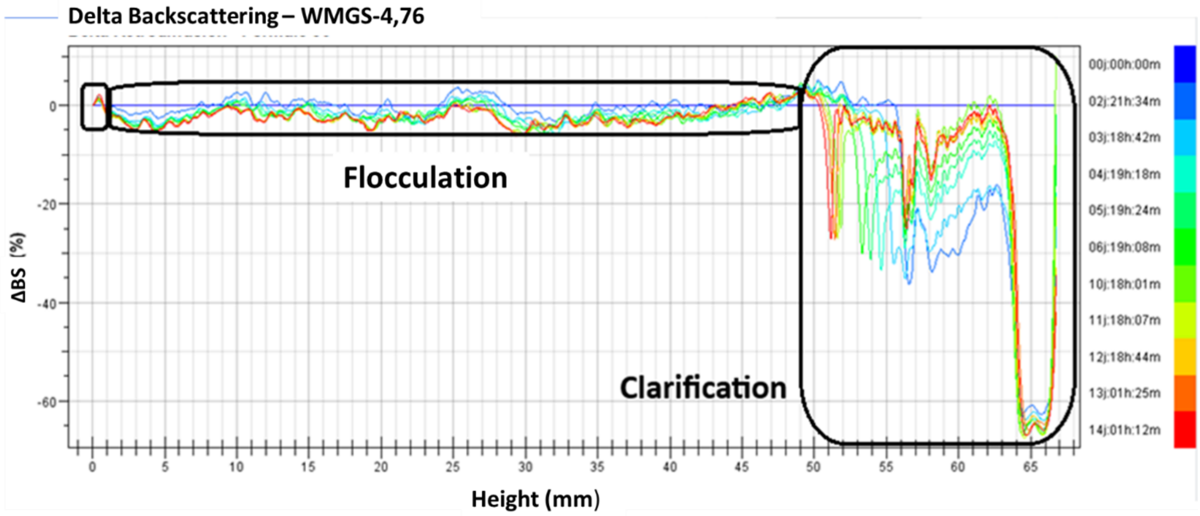
Delta Backscattering – WMGS-4,76
ΔBS (%)
Height (mm)
Flocculation
Clarification
00j:00h:00m
02j:21h:34m
03j:18h:42m
04j:19h:18m
05j:19h:24m
06j:19h:08m
10j:18h:01m
11j:18h:07m
12j:18h:44m
13j:01h:25m
14j:01h:12m

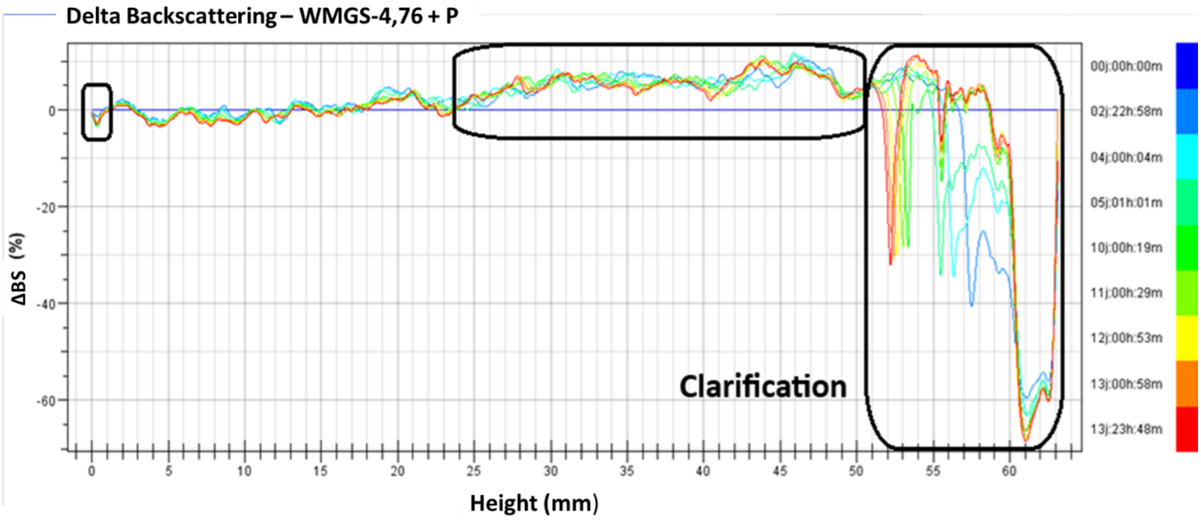
Delta Backscattering – WMGS-4,76 + P
ΔBS (%)
Height (mm)
Clarification
00j:00h:00m
02j:22h:58m
04j:00h:04m
05j:01h:01m
10j:00h:19m
11j:00h:29m
12j:00h:53m
13j:00h:58m
13j:23h:48m

Delta Backscattering – MC-0.40

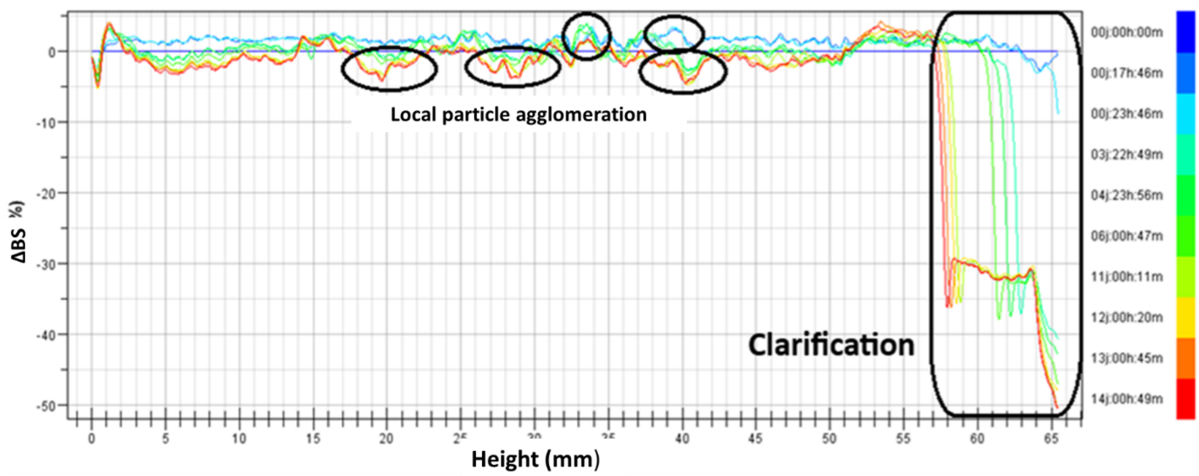

Delta Backscattering – MC-0.40 + P

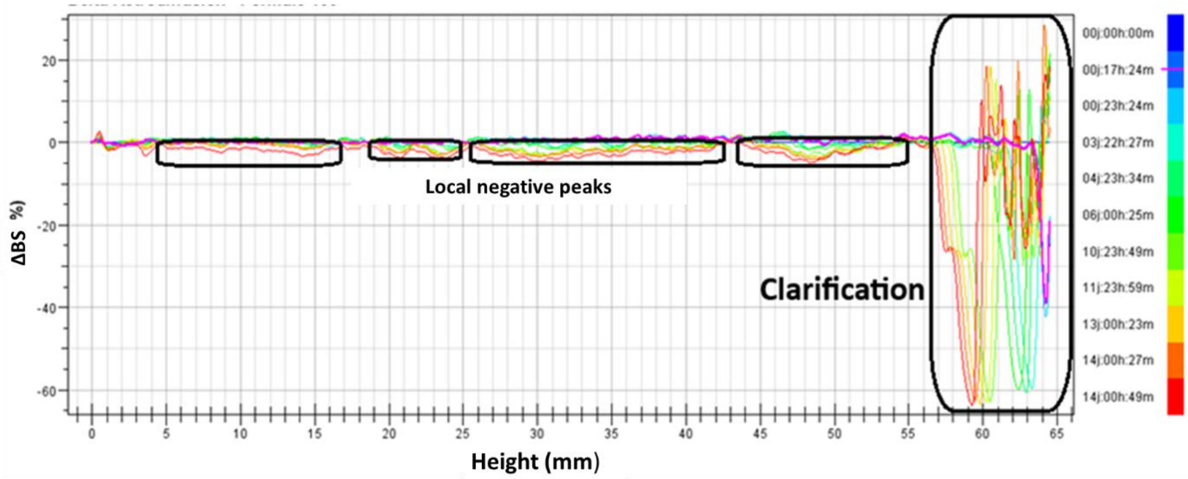

Delta Backscattering – PVA – 4.76

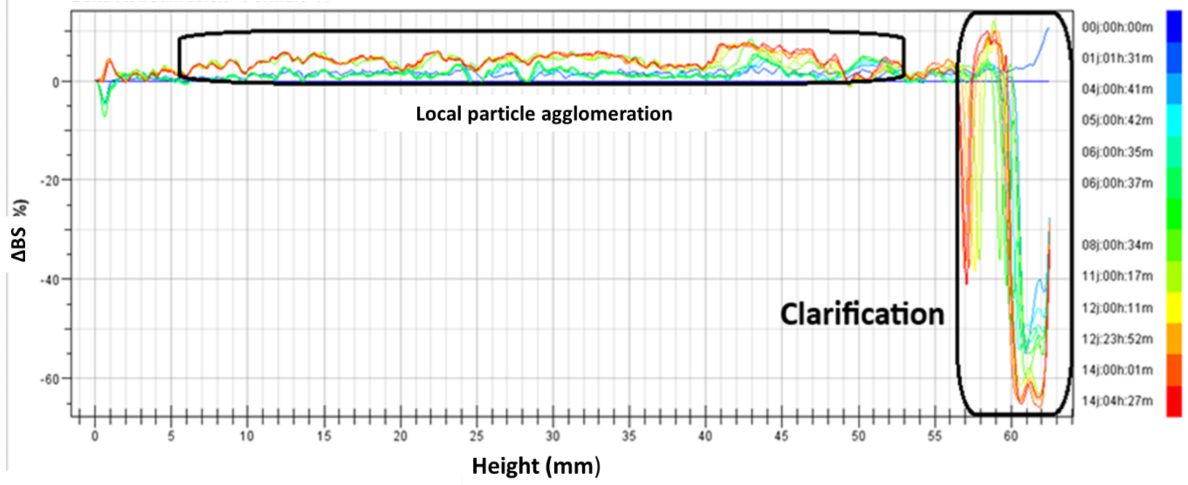

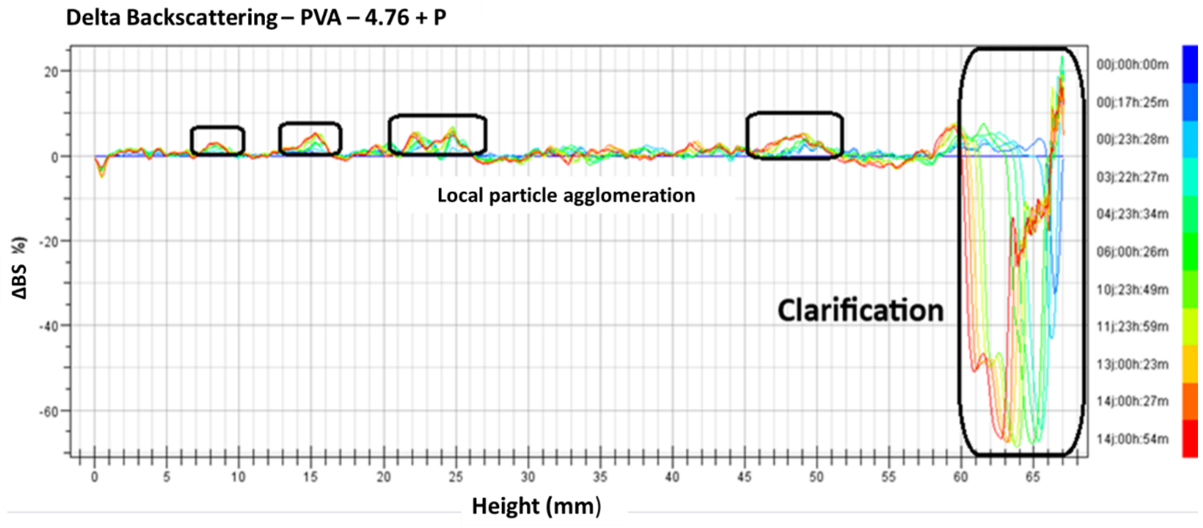

Delta Backscattering – PVA – 4.76 + P
Local particle agglomeration
Clarification
ΔBS (%)
Height (mm)
00j:00h:00m
00j:17h:25m
00j:23h:28m
03j:22h:27m
04j:23h:34m
06j:00h:26m
10j:23h:49m
11j:23h:59m
13j:00h:23m
14j:00h:27m
14j:00h:54m

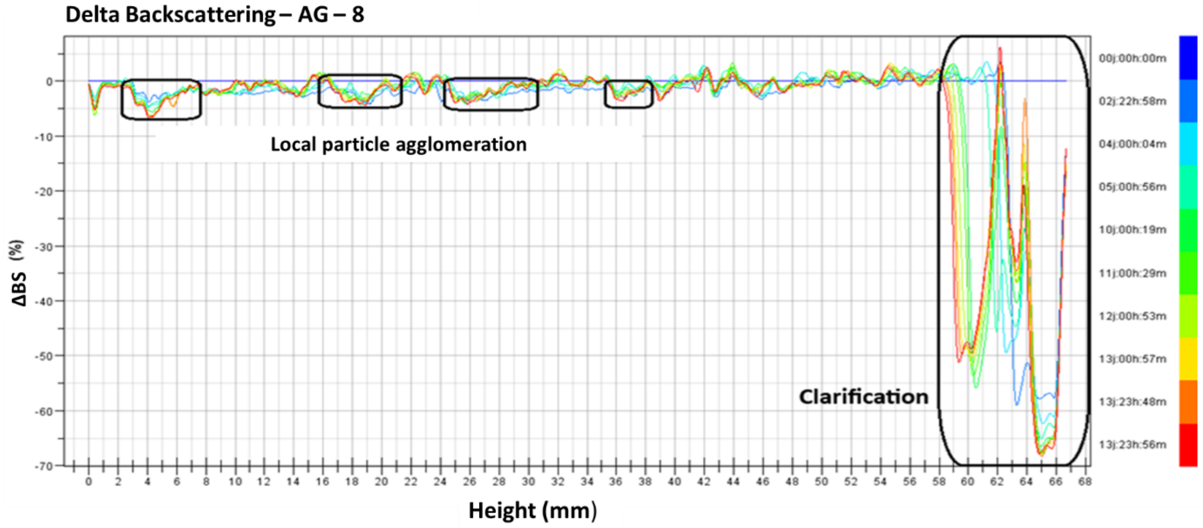

Delta Backscattering – AG – 8
Local particle agglomeration
Clarification
ΔBS (%)
Height (mm)
00j:00h:00m
02j:22h:58m
04j:00h:04m
05j:00h:56m
10j:00h:19m
11j:00h:29m
12j:00h:53m
13j:00h:57m
13j:23h:48m
13j:23h:56m

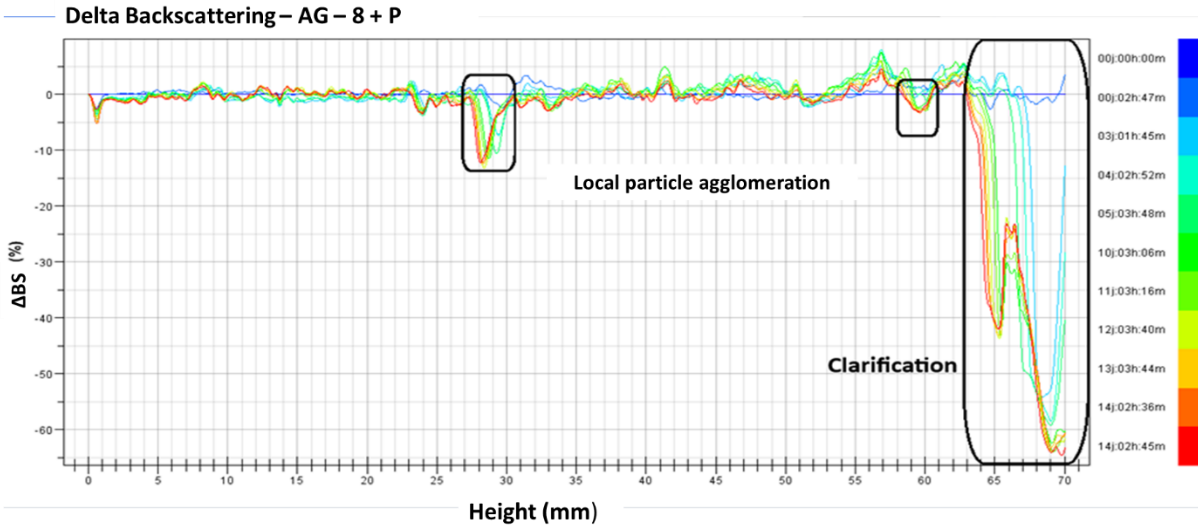

Delta Backscattering – AG – 8 + P
Local particle agglomeration
Clarification
ΔBS (%)
Height (mm)
00j:00h:00m
00j:02h:47m
03j:01h:45m
04j:02h:52m
05j:03h:48m
10j:03h:06m
11j:03h:16m
12j:03h:40m
13j:03h:44m
14j:02h:36m
14j:02h:45m

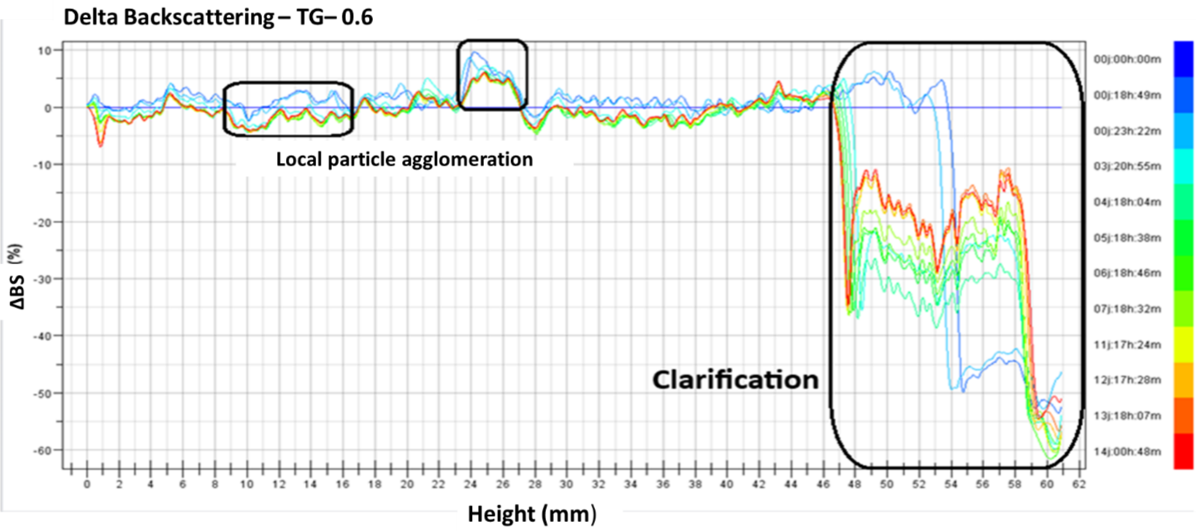
Delta Backscattering – TG– 0.6
ΔBS (%)
Height (mm)
Local particle agglomeration
Clarification
00j:00h:00m
00j:18h:49m
00j:23h:22m
03j:20h:55m
04j:18h:04m
05j:18h:38m
06j:18h:46m
07j:18h:32m
11j:17h:24m
12j:17h:28m
13j:18h:07m
14j:00h:48m

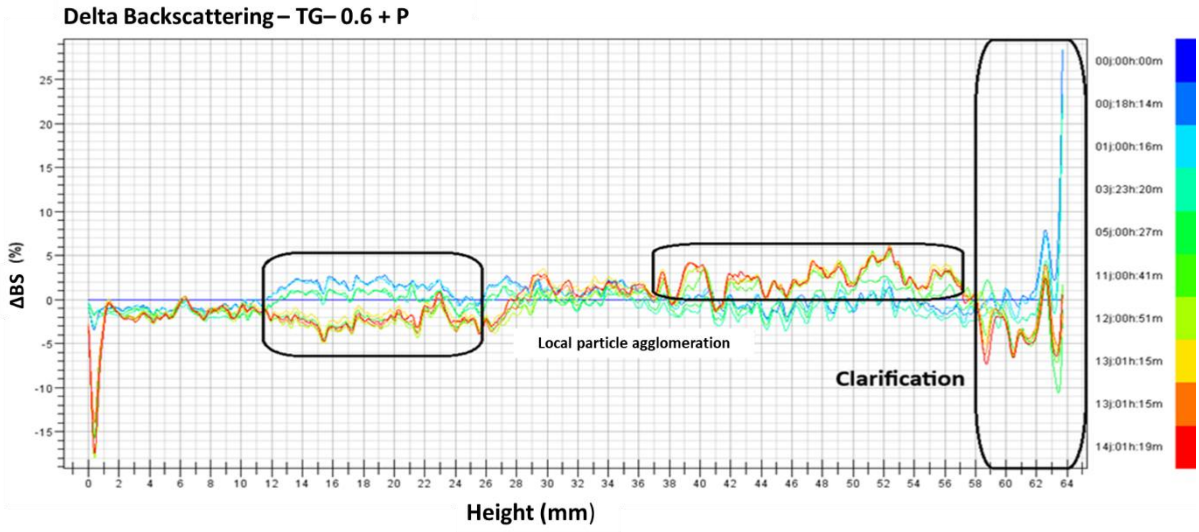
Delta Backscattering – TG– 0.6 + P
ΔBS (%)
Height (mm)
Local particle agglomeration
Clarification
00j:00h:00m
00j:18h:14m
01j:00h:16m
03j:23h:20m
05j:00h:27m
11j:00h:41m
12j:00h:51m
13j:01h:15m
13j:01h:15m
14j:01h:19m

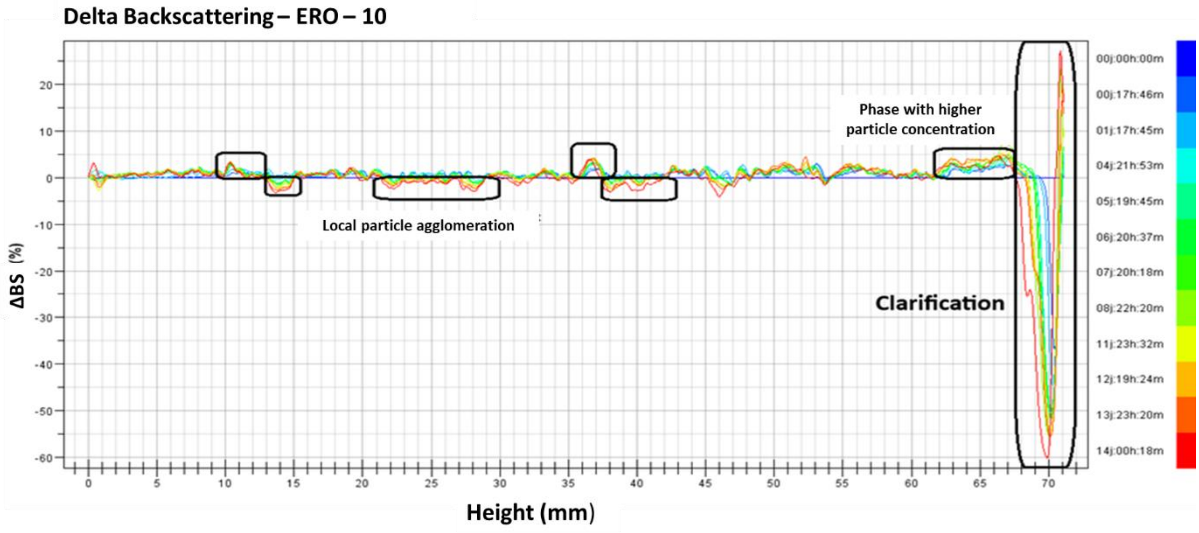
Delta Backscattering – ERO – 10
ΔBS (%)
Height (mm)
Local particle agglomeration
Phase with higher
particle concentration
Clarification
00j:00h:00m
00j:17h:46m
01j:17h:45m
04j:21h:53m
05j:19h:45m
06j:20h:37m
07j:20h:18m
08j:22h:20m
11j:23h:32m
12j:19h:24m
13j:23h:20m
14j:00h:18m

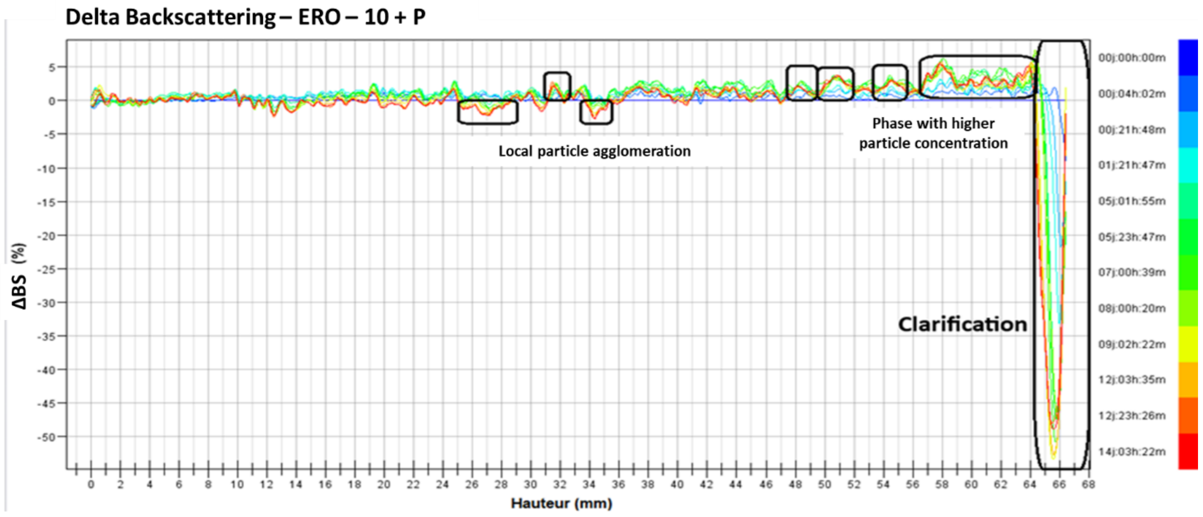

Figure S1 : Delta Backscattering profiles (ΔBS) of seed film-coatings formulas under study

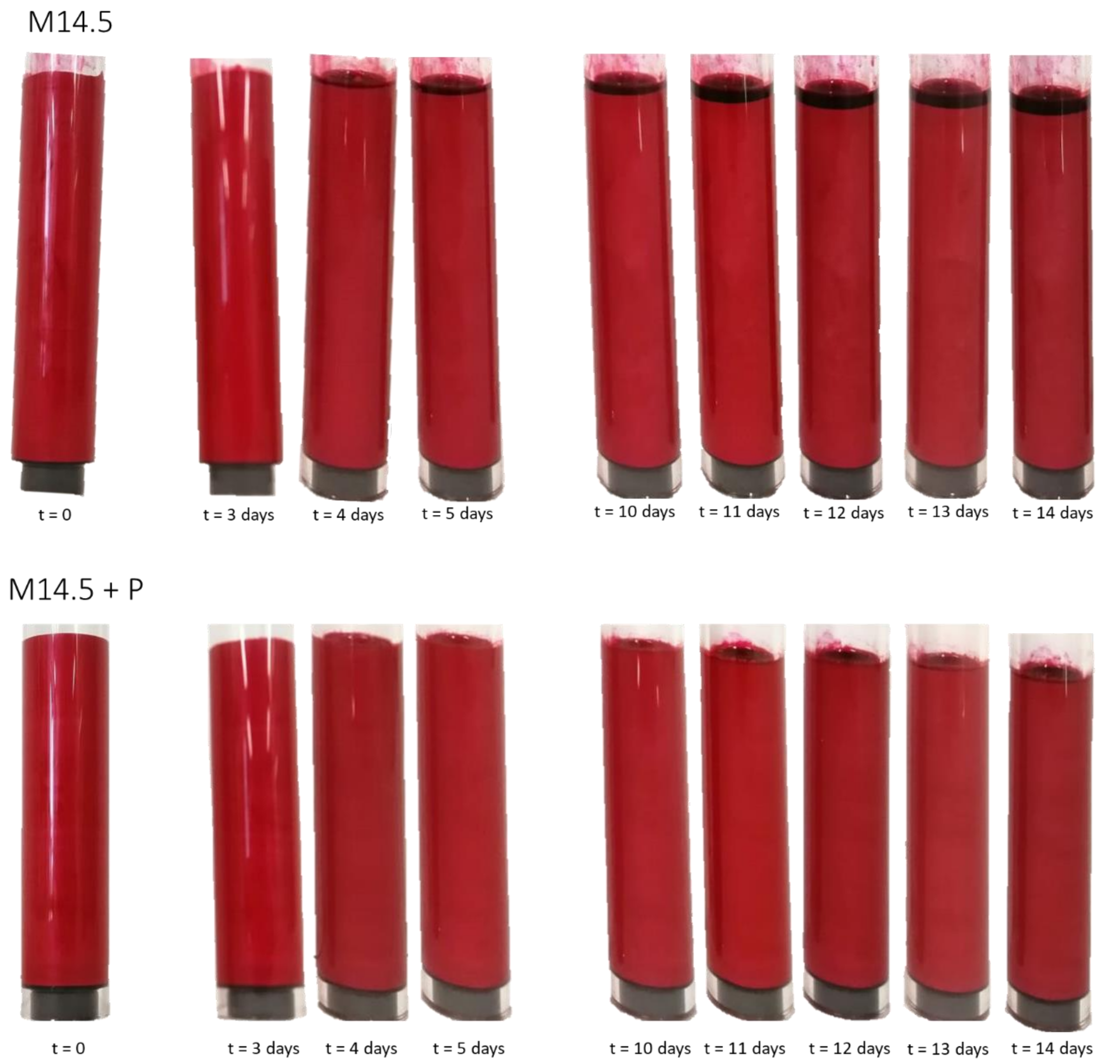

Figure S2 : Photographs of M14.5 (up) and M14.5 + P (down) during their accelerated aging.
